# The Impact of Knee Bending on the Superficial Femoral Artery and Popliteal Artery Morphology Before and After Endovascular Repair of Popliteal Aneurysm

**DOI:** 10.1177/15266028241245582

**Published:** 2024-08-06

**Authors:** Giovanni Spinella, Marco Magliocco, Bianca Pane, Giancarlo Salsano, Giuseppe Cittadini, Fabio Riccardo Pisa, Michele Conti

**Affiliations:** 1 Department of Surgical and Integrated Diagnostic Sciences, University of Genoa, Genoa, Italy; 2 Vascular and Endovascular Surgery, Istituto di Ricovero e Cura a Carattere Scientifico Ospedale Policlinico San Martino, Genoa, Italy; 3 Department of Experimental Medicine, University of Genoa, Genoa, Italy; 4 Department of Radiology, Istituto di Ricovero e Cura a Carattere Scientifico Ospedale Policlinico San Martino, Genoa, Italy; 5 Department of Civil Engineering and Architecture, University of Pavia, Pavia, Italy

**Keywords:** popliteal aneurysm, endovascular treatment, peripheral stenting, femoropopliteal segment, femoropopliteal deformations

## Abstract

**Objective:**

The aim of this study is to evaluate the deformations of the femoropopliteal (FP) arterial segment due to knee flexion in patients suffering from popliteal aneurysm before and after endovascular treatment (ET).

**Design and methods:**

Nine patients were prospectively evaluated. Pre-operative and post-operative computed tomography angiography (CTA) scans were performed on the leg of each patient in both a flexed and extended knee position. The images were employed to reconstruct the FP segment through segmentation and the resulting models were subsequently used to calculate the average diameter, length, and tortuosity of both the superficial femoral artery (SFA) and popliteal artery (PA). Furthermore, the overall PA tortuosity was decomposed into 2 components, ie, antero-posterior and lateral direction.

**Results:**

Following knee flexion, both arterial segments experienced shortening in the pre-operative and post-operative phases. Specifically, the SFA was shortened by 3.5% in pre (p<0.001) and 1.21% in post-stenting (p<0.001), while the PA was shortened by 4.8% (p<0.001) and 5.63% (p<0.001), respectively. Tortuosity significantly increased in all considered segments; in particular, in SFA there was a pre-intervention increase of 85.2% (p=0.002) and an increase of 100% post-intervention (p=0.004), whereas in the PA, there was an increase of 128.9% (p<0.001) and 254.8% (p<0.001), respectively. The only diameter variation occurred in the SFA pre-operatively with an increase of 11.9% (p=0.007). Tortuosity decomposition revealed significant differences between the 2 planes during the pre-operative and post-operative phases in both extended and flexed configurations, confirming a change in artery position and geometry due to treatment.

**Conclusions:**

Knee flexion induces arterial shortening and increased tortuosity in both the pre- and post-operative configuration. Stent placement does not induce significant geometric differences between pre-treatment and post-treatment. These results seem to indicate that the geometry of the covered stent is not affected by the flexion of the knee joint. Despite this, a more detailed analysis of arterial tortuosity showed a change in artery deformation following treatment.

**Clinical Impact:**

This study aimed to evaluate femoropopliteal arterial deformations in nine patients with popliteal aneurysm before and after endovascular treatment (ET) during knee flexion, using a standardized protocol for CTA acquisition and analysis. The result can be useful in procedure planning and have shown that the Viabahn stent used can adapt to the morphological variations induced by limb flexion. Consequently, device failure does not be attributed to stent compression but rather to other factors, such as alterations in hemodynamic and biomechanical forces on the implant due to the significant changes in tortuosity observed, or biological causes.

## Introduction

Popliteal artery aneurysms (PAAs) represent the most common peripheral aneurysms.^1^ Treatment aims to avoid ischemic complications that can lead to limb loss.^2,3^ Treatment options include open repair (OR) or endovascular treatment (ET).^4^ Nowadays, OR is reserved for patients with long life expectancy, whereas ET is indicated for patients with limited life expectancy when an autologous saphenous vein is available conversely.^5^

The issue associated with ET is that the covered stent is positioned at the level of the knee joint and is therefore subjected to repetitive deformations due to knee flexion.^6^ These significant load conditions can stimulate the development of thrombosis, restenosis, and endoleak, which promote mechanical failure of the device.^7^ Furthermore, ET should be considered in case of adequate proximal and distal landing zones, in the presence of at least 2 run-off vessels, and finally, in the absence of major tortuosity.^8^

It is therefore important to analyze this treatment option and, in particular, observe the behavior of the device in real “in vivo” conditions, which take into account, eg, the effect of musculature and daily deformations, in order to improve the outcomes of endovascular intervention. To the best of our knowledge, previous studies on the femoropopliteal (FP) artery deformations in vivo assessment were performed only in the post-operative condition. For example, Spinella et al^9^ recently analyzed the deformations induced by knee flexion in patients who underwent ET using computed tomography angiography (CTA). Consequently, studies that also analyze the pre-operative condition and make a comparison with the post-operative one have not yet been performed.

Given this, this study aims to evaluate the morphological changes of the FP segment due to knee flexion in patients with endovascularly treated PAAs using a standardized protocol for CTA acquisition in both straight- and bent-knee configurations before and after ET.

## Materials and Methods

Patients affected by a PAA who underwent endovascular repair treatment were recruited for a prospective imaging study to identify deformations induced by knee flexion and stent placement.

Signed consent was obtained for all patients and all procedures were performed by the Declaration of Helsinki. The study was approved by the Regional Ethics Committee (GR-2018-12368376).

Patients were selected to participate in the study based on specific criteria. One of the requirements for inclusion in this study was the availability of well-contrasted and noise-free scans of the knee in both extended and flexed positions, before and after surgery, in order to perform accurate analysis.

Inclusion criteria included individuals of both genders between the ages of 50 and 85 years who were affected by popliteal aneurysms. Exclusion criteria consisted of individuals who had undergone previous peripheral surgical or endovascular procedures, had contraindications to computed tomography (CT) scans, had medical conditions that limited their expected survival to less than 1 year, or were unable to provide informed consent.

Patients were eligible for ET of PAA in cases of adequate proximal and distal landing zone length (at least 15 mm) and the presence of at least 2 run-off vessels.

Viabahn stent-grafts (W.L. Gore & Associates, Flagstaff, Arizona, USA) were used for the ET of PAAs. As previously done in Spinella et al,^9^ and as reported by the guidelines,^5^ the devices were chosen in an appropriate number based on pre-operative CTA and in accordance with the length required to exclude the aneurysm. Briefly, the diameter of the covered stent was chosen in accordance with the nominal diameters of the distal landing zone and with an approximate 10% oversizing regarding the proximal landing zone. One more covered stent was used depending on the length to be covered and the proximal and distal diameters. The treatment was surgically performed on the superficial femoral artery (SFA) at the origin; the covered stent was deployed on a rigid guidewire. All patients received dual antiplatelet therapy during follow-up (FU).

### Image Acquisition

Two contrast-enhanced CTAs of the lower limb were acquired in each recruited patient: the first before ET and second at FU.

Two sets of images were obtained for both scans. In all acquisitions, the patient was placed in a supine position on the examination table. In the first set acquired, the knee was flexed at a 90° angle using a specific padded support positioned beneath the knee joint (bent-knee configuration). The second set was acquired with the knee joint extended (180°, straight-knee configuration).^9,10^

As in previous studies published by other research groups, an innovative CTA image acquisition protocol was used.^9^ This protocol enabled image analysis, allowing the evaluation of the characteristics of the SFA and the PA for both extended and flexed knees, minimizing the amount of contrast medium injected.

### Image Processing

Both prospective and retrospective CTA images were anonymized and transferred to a workstation (Windows 10 Home operating system, GPU: NVIDIA GeForge RTX 2080 Ti, CPU: Intel core i9-9900K 3.60 GHz, RAM: 32 GB) for image processing.

The image processing was similar to that described in previous works.^9–11^ The segmentation of all lower limb parts of interest was performed by ITK-Snap, an open-source software application specifically used to segment structures in 3-dimensional (3D) medical images.

As the images were acquired in different reference systems, a registration procedure was performed for each patient to bring all their acquisitions into the pre-operative extended knee reference system. This was achieved by utilizing the 3D model of the femur as it is a rigid structure that can be employed by the “vmtkicpregistration” module via a library of tools specifically conceived for the processing of vascular structures known as the Vascular Model Toolkit library (VMTK).^12^ This operation overlayed all the 3D models for each patient, enabling the division of the artery into the same regions of interest.

**Figure 1.**
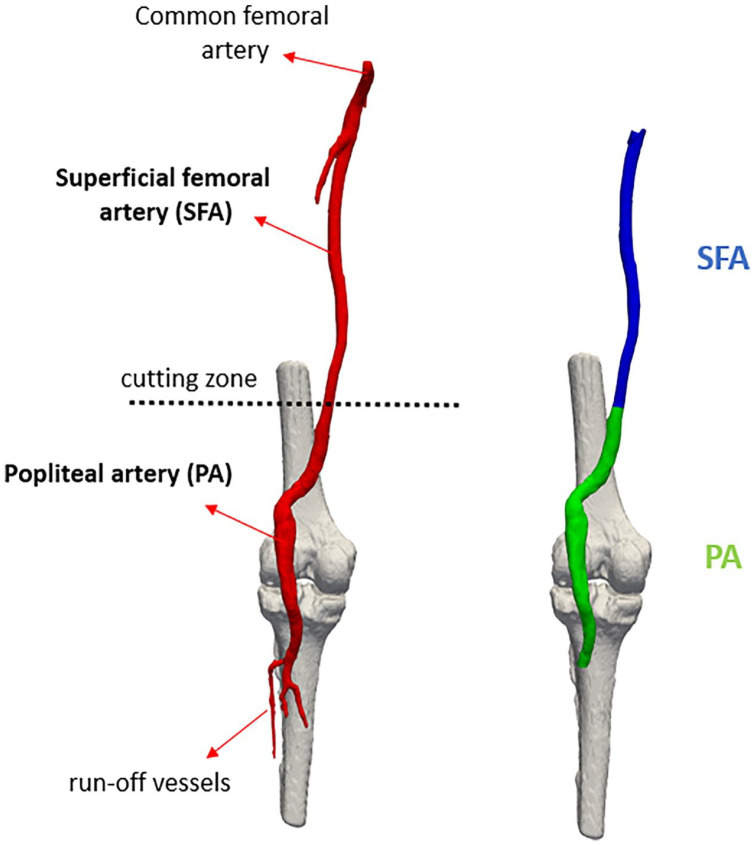
Image depicting the division in the segment of the superficial femoral artery and the segment of the popliteal artery. The cutting zone is located at the anatomical reference point used, namely the adductor hiatus.

Considering that the aim of the study was to observe how the geometry and hemodynamics of the femoropopliteal artery change after ET, the lumen was divided into 2 zones in both the pre-operative and post-operative cases, as shown in Figure 1. The first includes the SFA from the branch with the common femoral artery up to the popliteal artery (PA); whereas the second one includes the PA up to its branches (run-off vessels). The 3D models have been manipulated through ParaView (Kitware, Clifton Park, New York), an open-source application for data analysis and visualization.^13^

Using VMTK scripts, the centerline was automatically calculated, resampled, and then smoothed through a moving average filter for each vessel zone. The following parameters were subsequently calculated: centerline length, centerline global tortuosity, and mean diameter.

Starting from the vessel centerline and the lumen surface, equally spaced planes perpendicular to the centerline were automatically computed (vmtkcenterlinesections), and diameter measurements were taken on each transverse section. In particular, the cross-sectional sections were considered perfectly circular, and the diameter (D) was derived from the automatically returned area (A) value provided by VMTK using $D=2*\sqrt{\frac{A}{\pi }}$ . Subsequently, the mean diameter was calculated by averaging the diameter of each surface.

Tortuosity index (to describe vessel’s shape) was automatically computed by VMTK (just like the centerline length), and it was measured as a global parameter for each arterial segment. It was defined as T=L*D-1, where L was the centerline length and D was the distance between the centerline endpoints. Each of the computed parameters for each segment was compared between straight-leg and bent-leg configurations. Subsequently, to evaluate the endovascular procedure, comparisons were also made between the legs in the pre-operative configuration and the legs after the treatment. To determine the parameters variations, percentage variations were calculated using the formula

$$\%=\;\frac{\mathrm{median}\_\mathrm{straight}\;-\;\mathrm{median}\_\mathrm{bent}}{\mathrm{median}\_\mathrm{straigtht}}*100$$

**Figure 2.**
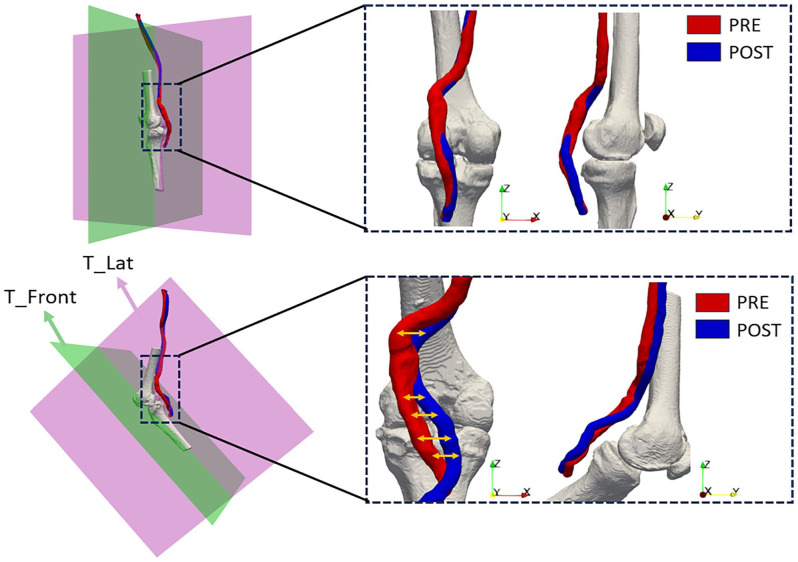
Image depicting the decomposition of tortuosity on the antero-posterior plane (green) and the lateral plane (purple).

Furthermore, to better understand the deformation modes of the popliteal artery (which undergoes greater deformation as it is located at the knee joint), it was decided to project the centerline of the segment into 2 planes that reference an antero-posterior direction (T_Front) and a lateral direction (T_Lat), as shown in Figure 2. The planes were created using ParaView, both for the knee in an extended configuration and the bent configuration. The tibia was used as a reference for their creation; in particular, planes in its orthogonal direction were used. For each point of the centerline, orthogonal projection onto the planes was then performed using MATLAB R2022b (The Mathworks, Natick, Massachusetts, USA) software and the tortuosity was subsequently derived by calculating the length of the projection line.

### Statistical Analysis

Continuous variables are expressed as median and interquartile range (IQR). Differences between knee configurations were analyzed using a paired-sample *t*-test in the case of normally distributed data (normality assumption was tested using the Kolmogorov-Smirnov test). If the assumption of normality was not met, the paired Wilcoxon signed-rank test was performed. p-values<0.05 were considered statistically significant. All statistical analyses were performed using GraphPad Prism version 8.0.0 (GraphPad Software, San Diego, California).

## Results

This study included 9 patients who underwent a PAA endovascular procedure. The patients were prospectively enrolled in the study (between September 2019 and April 2022) according to inclusion and exclusion criteria.

Their characteristics, risk factors, detail on stent number, diameter, and length for each patient are reported in Table 1.

**Table 1.** Characteristic of the Stent.

Patient	No. of stent	Diameter (mm)	Length (mm)	Stent position
1	1	8	250	SFA/PA
	2	7	100	
2	1	11	100	PA
3	1	7	100	PA
	2	6	150	
4	1	9	50	PA
	2	7	150	
5	1	8	10	SFA/PA
6	1	10	150	SFA/PA
	2	11	100	
	3	13	100	
7	1	13	50	SFA/PA
	2	13	100	
	3	13	100	
	4	13	100	
8	1	10	100	PA
	2	11	100	
9	1	7	150	SFA/PA
	2	9	150	

All patients were male, and the median age was 77 years (IQR=3), and the median FU time was 8.12 months (IQR=4.83). A linear regression analysis was conducted to assess the relationship between the FU time and the variations in each measured parameter. The result was not statistically significant (Diameter SFA: R^2^=0.4, p=0.04; Diameter PA: R^2^=0.16, p=0.3; Length SFA: R^2^=0.01, p=0.8; Length PA: R^2^=0.2, p=0.18; Tortuosity SFA: R^2^=0.023, p=0.7; Tortuosity PA: R^2^=0.16, p=0.28); therefore, the variations were not influenced by the FU period.

Arterial diameter, length, and tortuosity for each zone (SFA and PA) in straight-leg and bent-leg configurations are reported in Table 2.

**Table 2.** Geometric Analysis Results.

Time	Configuration	SFA	PA
**DIAMETER (mm)**			
PRE	Straight	7.93 (3.5)	9.06 (3.82)
	Bent	9 (3.86)	9 (4.7)
FU1	Straight	7.59 (3.17)	6.8 (3.87)
	Bent	8.52 (4.4)	6.5 (3.81)
**LENGTH (mm)**			
PRE	Straight	291.14 (47.81)	218.31 (42)
	Bent	280.95 (46.97)	207.83 (36.28)
FU1	Straight	291.03 (45.1)	208.17 (34.1)
	Bent	287.5 (45.1)	196.45 (39.98)
**TORTUOSITY (−)**			
PRE	Straight	0.027 (0.01)	0.083 (0.059)
	Bent	0.05 (0.025)	0.19 (0.12)
FU1	Straight	0.03 (0.011)	0.062 (0.043)
	Bent	0.06 (0.04)	0.22 (0.07)

The results of geometric changes between the extended and bent-knee configurations in the pre-operative time point showed a statistically significant variation in diameter in the SFA segment, specifically, an increase of 11.9% (p=0.007) from 7.93 to 9 mm due to the knee flexion. No variation in diameter was observed for PA.

**Figure 3.**
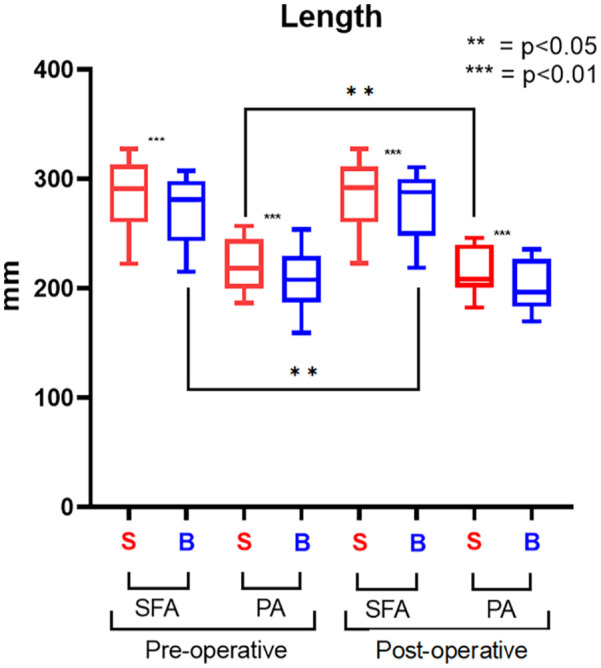
Length results. The straight-leg configuration (S) is represented in red; the bent-leg configuration (B) is represented in blue.

There were statistically significant variations in length observed in both the SFA and in the PA. Specifically, the arterial length decreased following knee flexion, as shown in Figure 3.

In SFA, the artery shortened by 3.5% (p<0.001) from 291.14 (IQR=47.81) to 280.95 (IQR=46.97), whereas in the PA, the length decreased 4.8% (p<0.001) from 218.31 (IQR=42) to 207.83 (IQR=36.28).

**Figure 4.**
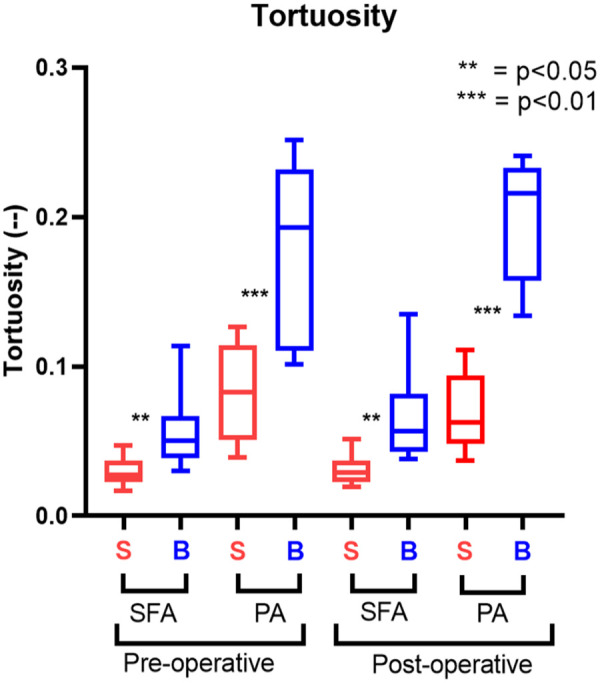
Tortuosity results. The straight-leg configuration (S) is represented in red; the bent-leg configuration (B) is represented in blue.

The tortuosity significantly increased following knee flexion in both the SFA and PA, as shown in Figure 4. In SFA, it increased 85.2% (p=0.002), changing from 0.027 (IQR=0.01) to 0.05 (IQR=0.025), and in PA of 128.9% (p<0.001) from 0.083 (IQR=0.059) to 0.19 (IQR=0.12).

After stent placement, there were no significant variations in diameter and it remained constant during knee flexion. The arterial length decreased (Figure 3) particularly in SFA where the artery shortened by 1.21% (p<0.001) from 291.03 (IQR=45.1) to 287.5 (IQR=45.1), whereas the PA length decreased by 5.63% (p<0.001) from 208.17 (IQR=34.1) to 196.45 (IQR=39.98).

Similar to the pre-operative phase, post-treatment, an increase in tortuosity was observed (Figure 4). Specifically, a 100% increase was noted in SFA (p=0.004, 0.03 [IQR=0.011] to 0.06 [IQR=0.04]) and a 254.8% increase in the PA (p<0.001, 0.062 [IQR=0.043] to 0.22 [IQR=0.07]).

Finally, the results obtained in the pre-operative phase were compared to those after ET. Regarding the diameter variations, there was a significant reduction in PA after stent apposition both in straight and bent knees. The reduction was approximately 27%, from 9 to about 6.5 mm in both cases. No diameter variation was observed in SFA.

There were no significant changes in arterial length, except for the SFA in the flexed knee (p=0.017) from 280.95 (IQR=46.97) to 287.5 (IQR=45.1) and the PA in the extended knee (p=0.041) from 218.31 (IQR=42) to 208.17 (IQR=34.1). There were not any changes in tortuosity between the pre-phase and post-phases.

**Figure 5.**
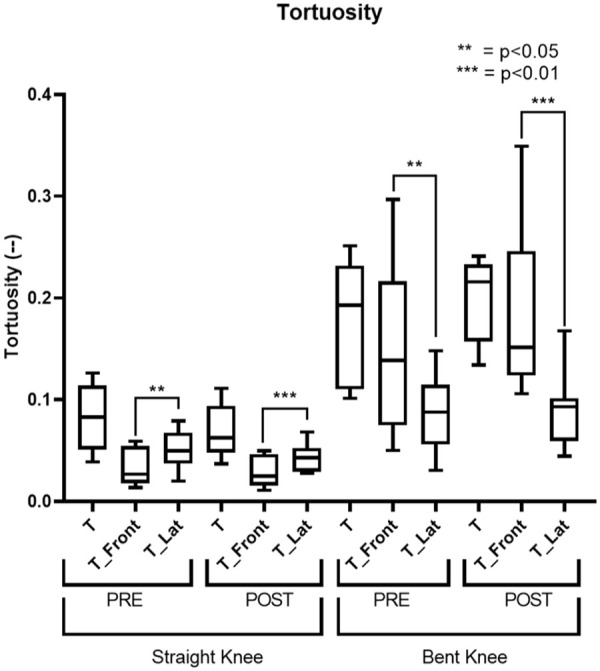
This figure shows the results related to the decomposition of tortuosity. T represents the overall tortuosity, T_Front indicates the component projected onto the antero-posterior plane, and T_Lat the component projected onto the lateral plane.

The decomposition of tortuosity showed significant differences between the antero-posterior plane (T_Front) and the lateral plane (T_Lat) in each segment, as shown in Figure 5. The results are reported in Table 3. In the straight knee, tortuosity increased from 0.026 (IQR=0.036) on the “T_Front” to 0.05 (IQR=0.027) on the “T_Lat” plane in the pre-operative phase (p=0.02). After the treatment, it changed from 0.025 (IQR=0.03) to 0.043 (IQR=0.022) (p=0.008). However, in the flexed knee, tortuosity was higher on the “T_Front,” with values of 0.14 (IQR=0.12) in the pre-operative and 0.15 (IQR=0.1) in the post-operative phase, compared with 0.087 (IQR=0.052) and 0.093 (IQR=0.04) on the “T_Lat” plane, (p=0.02 and p=0.016), respectively.

**Table 3.** Decomposition of Tortuosity Results.

	PRE		POST	
	T_Front	T_Lat	T_Front	T_Lat
**Straight Leg**	0.026 (0.036)	0.05 (0.027)	0.025 (0.03)	0.043 (0.022)
**Bent leg**	0.14 (0.12)	0.087 (0.052)	0.15 (0.10)	0.093 (0.04)

## Discussions

Pre-operative and post-operative in vivo deformation conditions have never been compared in aneurysmal patients. For this reason, in this study, arterial deformations were analyzed in 9 patients who underwent endovascular procedures for PAAs by analyzing medical images. For each patient, a comparison of parameters (length, tortuosity, and diameter) was made between the extended knee and the flexed knee in both the pre-operative and post-operative time points. Subsequently, a comparison was also made between the pre-operative and post-operative legs to understand the effect of stent placement in the artery.

Considering pre-operative changes of the arterial shape due to knee flexion, our results showed a significant increase in the diameter of the SFA segment, arterial shortening of both the popliteal segment and the SFA with a significant increase in tortuosity. Currently, ET planning is based on a CT scan taken with the knee extended, which assumes that the knee area is static. Our study confirms that the femoral-popliteal area, associated with the knee joint, is dynamic and subject to configuration changes^14^ and biomechanical actions.^15^ Such results suggest that by analyzing the geometry of the flexed knee, we can obtain more accurate information for ET planning, which may lead to better FU results.

After the stent placement, the artery continued to shorten upon knee flexion and became more tortuous, but there was no change in its diameter. After comparing the pre-operative and post-operative conditions, it was found that the diameter of the popliteal artery decreased significantly. However, there were no significant changes in arterial shortening and variation in tortuosity after stent placement compared with the pre-operative time. Nevertheless, the decomposition of tortuosity showed significant results.

Both the popliteal and SFA segments shorten during knee flexion both pre-operatively and post-operatively. The greatest shortening is related to the PA tract, which is located in the most mobile segment of the femoropopliteal axis, as previously demonstrated on ex vivo models^16–18^ and on patients with occlusive disease.^19,20^ The changes in shortening following stent placement did not show significant variations. Superficial femoral artery decreased from 3.5% to 1.21%, whereas the popliteal artery increased from 4.8% to 5.63%. In the pre-operative instant, a variation in the diameter of the SFA was observed; this variation occurs only in this arterial segment and not in the popliteal area, as the presence of the aneurysmal sac already dilates the popliteal artery.

Our results showed that the diameter of the popliteal artery did not change after bending the knee. This aspect could be taken into account in order to avoid excessive oversizing in the distal landing zone, assuming that the covered stent, placed with a lower oversize, could be less constrained and could mimic the geometric changes related to the flexion of the knee. Indeed, some authors have reported that greater oversize may be a risk factor for occlusion or distal edge restenosis during FU.^21^

The change in diameter of the SFA was approximately 11% and is probably due to the vessel-compensating effect of the shortening observed during knee flexion. This information could be useful in planning the ET; in fact, it is necessary to pay particular attention in choosing the diameter of the stent to be positioned for the proximal landing zone, which many times falls at the level of the distal SFA. A higher oversize for the proximal landing zone could be considered to avoid endoleak or stent migration related to the biomechanical stresses of knee flexion.

After stent placement, there was no significant difference in artery diameter; however, comparing the arterial section before and after the intervention, it decreased by approximately 27%. This is not due to a morphological variation but only to the treatment result (excluding the aneurysmal sac).

Zaghloul et al^22^ indicate that in the case of ET with stent-grafts extending below the knee joint to the run-off vessels, the risk of intervention failure increases, reporting a total of 19 occlusions out of 117 positioned stents. However, our results did not show significant diameter variations in the popliteal artery; consequently, occlusion seems not due to stent compression. Instead, it could be attributed, eg, to the below reported tortuosity variations, which may lead to stent kinking and alterations in hemodynamic/biomechanical forces affecting the device or to other causes of a different nature yet to be investigated.

Following limb flexion, the tortuosity of both arteries increased significantly. The placement of the stent increased the tortuosity of the artery more but the change was not statistically significant. These results suggest that the Viabahn stent can accommodate arteria morphological variations induced by knee flexion, as evidenced by the unchanged global parameters (length, tortuosity, diameter). The capabilities of the Viabahn stent had already been demonstrated by a previous study conducted on ex vivo models^23^ and by mechanical tests performed in laboratory.^24^

However, the results obtained from the decomposition of tortuosity showed differences in the way the artery deforms and positions following treatment and bending. In fact, it was already known that the position of the artery changed following knee flexion, as also illustrated by Yoo et al^25^ and Takeda et al,^26^ but it had not yet been observed how the morphology varies following stent placement.

This morphological difference could cause stent failures, as it changes the biomechanical conditions to which the artery is subjected and will be investigated in future studies through the implementation of patient-specific biomechanical finite element simulations. One of the most significant variations we have observed relates to tortuosity. In our analysis, tortuosity always increases after knee flexion. This aspect should be better investigated by computational fluid dynamics simulations to understand whether tortuosity variations are related to flow alterations promoting stent occlusion during FU. In this context, we have recently shown that stent flow changes occur and that over time these changes can lead to formation of an endoluminal thrombus wall that may account for stent occlusion at FU.^10,27^ Although the analysis method is similar to a previous one, the study in question involved retrospectively enrolled patients treated between 2013 and 2017.^9^ Furthermore, the first analysis was performed only on post-operative images. In this article, however, prospective enrollment of new patients was performed, and the analysis was conducted on both pre-operative and post-operative CTA, allowing for the examination of differences related to the stent graft deployment.

### Limitations

The total number of patients (9) may be a limitation to the interpretation of the study results. Nevertheless, the first aspect to be taken into consideration is that the IQR of the evaluated parameters was stable. Therefore, the results derived are reliable, even though obtained from few patients.

Furthermore, another aspect to take into account is that the data refer to geometric analysis obtained from CT analysis. In this routine setting, no pre-treatment flexed and extended knee acquisitions were performed and neither were any FU CTs. This aspect, therefore, currently makes our evaluations unique in the literature.

Another limitation to consider is that the 9 patients included in the study did not all undergo treatment with the same number of stents placed in the same location. Indeed, as the lesions varied in length among different patients and considering that not all combinations of lengths and diameters for implantable devices were available on the market, in some cases, it was necessary to position multiple overlapping stents. However, the regions of overlap affected the rigidity of the implant which, consequently, responded differently to the stresses imposed by knee flexion.

Future studies should investigate how arterial deformation and biomechanics of the devices vary in response to the placement of multiple overlapping stents compared with single ones. Furthermore, in this study deformation was studied at a fixed angle of 90 degrees. However, in reality, the maximum deformation occurs at higher angles, in what is commonly called the “gardening” position. Unfortunately, reproducing the same position in elderly and ill subjects within a CT is not possible. Nevertheless, we observed significant changes in many parameters evaluated with 90° bending. Future studies can be carried out with different degrees of flexion to evaluate the impact of flexion.

## Conclusions

In this study, in vivo deformations of the FP arterial segment were investigated in patients with PA who were treated endovascularly through the placement of Viabahn stents. The results demonstrated arterial shortening following knee flexion and increased tortuosity both in the pre-operative and FU phases. Regarding the diameter, there were no significant variations, except in the pre-operative SFA where it increased following flexion.

The differences in global parameters between pre-intervention and post-intervention were not significant; therefore, it is possible to say that the stent is not influenced by knee flexion in geometric terms. However, the arterial configuration changed following the procedure, as demonstrated by the results of its decomposition on the 2 anatomical planes (lateral and frontal). These results can be utilized in the future to perform computational simulations in order to understand how this morphological variation affects device failure.

## Supplemental Material

sj-docx-1-jet-10.1177_15266028241245582 – Supplemental material for The Impact of Knee Bending on the Superficial Femoral Artery and Popliteal Artery Morphology Before and After Endovascular Repair of Popliteal Aneurysm Supplemental material, sj-docx-1-jet-10.1177_15266028241245582 for The Impact of Knee Bending on the Superficial Femoral Artery and Popliteal Artery Morphology Before and After Endovascular Repair of Popliteal Aneurysm by Giovanni Spinella, Marco Magliocco, Bianca Pane, Giancarlo Salsano, Giuseppe Cittadini, Fabio Riccardo Pisa and Michele Conti in Journal of Endovascular Therapy
