# Supplementary material for: The Impact of Knee Bending on the Superficial Femoral Artery and Popliteal Artery Morphology Before and After Endovascular Repair of Popliteal Aneurysm
Source: J Endovasc Ther. 2024 Aug 6;33(1):203–12. doi: 10.1177/15266028241245582 (PMC12804411; doi:10.1177/15266028241245582)
Supplement: sj-docx-1-jet-10.1177_15266028241245582 – Supplemental material for The Impact of Knee Bending on the Superficial Femoral Artery and Popliteal Artery Morphology Before and After Endovascular Repair of Popliteal Aneurysm [file sj-docx-1-jet-10.1177_15266028241245582.docx]

**The impact of knee bending on the superficial femoral artery and popliteal artery morphology before and after endovascular repair of popliteal aneurysm**

**Supplementary table S1.** Population statistics.

|  | Total |
| --- | --- |
| Patients | 9 |
| Median Age | 77 (3) |
| Median Follow-up (Months) | 8.12 (4.83) |
| Laterality |  |
| Right | 6 |
| Left | 3 |
| Cardiovascular Risk Factors |  |
| Smoke | 3 |
| Hypertension | 5 |
| Diabetes | 0 |
| Alcohol | 0 |
| Hypercholesterolemia | 2 |
| BPCO | 1 |
| Coronary artery disease | 0 |
| Aortic segment atherosclerosis | 2 |
| Aneurysm/dissection aortic | 3 |
| Previous vascular operation | 7 |
